# Epileptic Encephalopathy Related to CAD Deleterious Variants—A Case Series

**DOI:** 10.3390/diseases13040091

**Published:** 2025-03-22

**Authors:** Adelina Glangher, Magdalena Budișteanu, Diana Bârcă, Dana Șurlică, Florentina Ionela Lincă, Doina Ioana, Laurentiu-Camil Bohîlțea, Ina-Ofelia Focșa, Catrinel Iliescu

**Affiliations:** 1 Psychiatry Research Laboratory, Prof. Dr. Alex. Obregia Clinical Hospital of Psychiatry, 041914 Bucharest, Romania; 2 Department of Genetics, Faculty of Medicine, Titu Maiorescu University, 031593 Bucharest, Romania; 3 Medical Genetics Laboratory, Victor Babes National Institute of Pathology, 050096 Bucharest, Romania; 4 Pediatric Neurology Department, Prof. Dr. Alex. Obregia Clinical Hospital of Psychiatry, 041914 Bucharest, Romania; 5 Pediatric Neurology Department, University of Medicine and Pharmacy Carol Davila, 020021 Bucharest, Romania; 6 Department of Special Psychopedagogy, Faculty of Psychology and Educational Sciences, University of Bucharest, 0506578 Bucharest, Romania; 7 Department of Medical Genetics, Faculty of Medicine, University of Medicine and Pharmacy Carol Davila, 020021 Bucharest, Romania

**Keywords:** CAD, EIEE-50, severe metabolic disorder, treatable

## Abstract

Background: Epilepsy, particularly early-onset and drug-resistant forms, presents a significant challenge in pediatric neurology. Inborn errors of metabolism are increasingly recognized as important contributors to these types of epilepsy. Timely diagnosis and treatment are crucial in preventing irreversible metabolic damage and improving clinical outcomes in CAD deficiency. This condition is a progressive and severe metabolic disorder caused by biallelic deleterious variants in *CAD* gene, and is characterized by long seizures, psychomotor regression, and dyserythropoietic anemia. Methods: In this paper, we present four new cases of EIEE-50, emphasizing the importance of early, specific therapeutic interventions. Results: Oral uridine 100 mg/kg/day was administrated with improvement of motor and cognitive function as well as immediate seizures control. Conclusions: Our findings underscore the potential for improved outcomes of EIEE-50 trought timely diagnosis and targeted treatment strategies, reinforcing the role of uridine supplementation as a promising therapeutic approach.

## 1. Introduction

Epilepsy, particularly early-onset and drug-resistant forms, presents a significant challenge in pediatric neurology. Seizures control is closely related to the genetic etiology and is crucial for the favorable outcome of the disorder. Thus, an early genetic diagnosis is mandatory for a proper management of the patients in the term of improving the quality of life [1,2,3]. Inborn errors of metabolism are increasingly recognized as important contributors to these types of epilepsy. Timely diagnosis and adequate treatment can prevent irreversible metabolic damage [4,5]. Among these metabolic disorders, CAD (Carbamoyl-Phosphate Synthetase 2, Aspartate Transcarbamylase, and Dihydroorotase) deficiency, also known as early infantile epileptic encephalopathy-50 (EIEE-50) or alternative title, developmental and epileptic encephalopathy 50 (DEE50, #OMIM 61645), stands out due to its severe and progressive nature [6].

CAD deficiency is an autosomal recessive, progressive, and severe metabolic disorder characterized by long seizures, psychomotor regression, and dyserythropoietic anemia [7]. The metabolic pathway regulated by *CAD* gene (OMIM# 114010) is essential for nucleotide homeostasis, cell growth, and proliferation. The *CAD* gene, located on chromosome 2p23.3, encodes a multifunctional protein involved in the first three steps of de novo pyrimidine synthesis, which is crucial for RNA and DNA synthesis [8,9]. The final product of this pathway, uridine monophosphate (UMP), is the substrate for all cellular pyrimidines. The main steps of UMP synthesis consist in

1. Enzyme Complex Formation: Glutaminase (GLN), carbamoyl phosphate synthetase (CPS), aspartate transcarbamoylase (ATC), and dihydroorotase (DHO) merge into the CAD protein complex [10].
2. Mitochondrial Catalyzation: The CAD protein is catalyzed by dihydroorotate dehydrogenase (DHODH) in the mitochondria [11].
3. Final UMP Synthesis: UMP synthase (UMPS), a bifunctional enzyme with orotate phosphoribosyl transferase (OPRT) and orotidine decarboxylase (ODC) activities, completes the synthesis of UMP [12].

**Figure 1:**
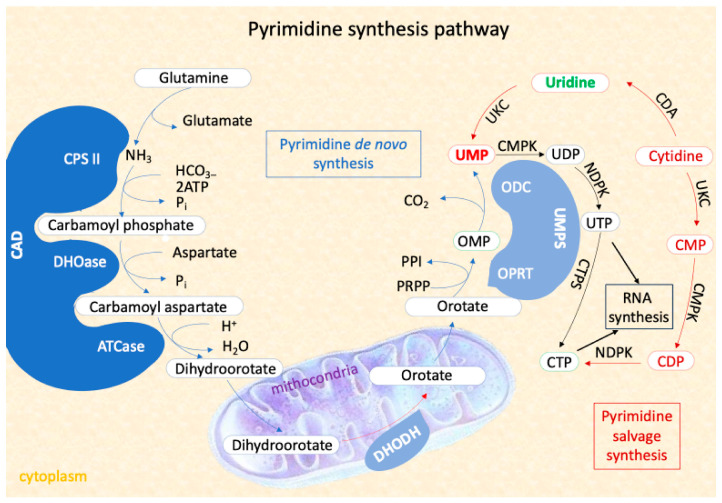
The Pyrimidine synthesis pathway including the two process of pyrimidine nucleotides synthesis: the novo pathway and the salvage pathway. The first three steps of the de novo pathway are catharized by trifunctional enzyme CAD.

Alternatively, UMP can be synthesized from uridine through salvage pathways, using intracellular nucleic acid degradation product or extracellular nucleoside, which can compensate for deficiencies and alter disease prognosis (Figure 1).

The *CAD* gene is large and complex, consisting of 44 exons that encode a protein with multiple enzymatic activities [13,14,15]. Deleterious variants in *CAD* disrupt the synthesis of pyrimidines, leading to cellular and metabolic dysfunctions that are clinically reflected in progressive neurological deterioration, drug-resistant seizures and anemia. Over 80 deleterious sites have been identified so far, adding to the complexity of diagnosing and understanding the full spectrum of the disorder. The variants associated with CAD deficiency can include missense, nonsense, and splice site, more than 75% of the variants being missense. These variants can lead to a complete loss of function or a significantly reduced function of the CAD protein [16,17]. The exact nature of the mutation can influence the severity and the specific symptoms of the disorder. Due to the gene’s large size and the nonspecific clinical presentation, genetic testing for CAD deficiency often involves whole-exome sequencing or targeted gene panels that include the *CAD* gene. Early diagnosis is challenging but critical for initiating appropriate treatment and improving outcomes [18,19]. Over the past eight years, studies have suggested that uridine therapy may mitigate symptoms by bypassing the defected de novo pyrimidine synthesis pathway [19,20,21].

## 2. Materials and Methods

EIEE-50 is notoriously difficult to diagnose due to its nonspecific clinical presentation. Key symptoms primarily affect the neurological system, including global developmental delay, drug-resistant epilepsy with multiple seizure types (tonic, generalized tonic-clonic, behavioral arrest, focal motor seizures), ataxia, tremor, hypotonia, brain atrophy, cognitive deficiency, and autism spectrum disorder [22]. In addition, dyserythropoietic anemia with anisopoikilocytosis ascertained by peripheral blood smear has been described [23]. Gastrointestinal complications such as feeding difficulties, gastroesophageal reflux, and recurrent vomiting are also noted. Although dysmorphic features are reported, a specific facial phenotype has not been described. Other less affected systems include the skeletal, cardiac, and visual systems [24]. The natural course of the disease is with progressive deterioration and often is fatal [6,22,25].

In this paper, we present four new cases of EIEE-50, analyzed in the context of previous reported findings, to further elucidate the clinical spectrum and response to treatment, emphasizing the importance of early, specific therapeutic interventions. Our findings underscore the potential for improved outcomes with timely and targeted treatment strategies.

## 3. Results

### 3.1. Case Series

Case 1 is a 5-year-old girl, with unremarkable family history, uneventful pregnancy and birth. She was first admitted in our clinic at 1 year and 11 months for global developmental delay and febrile focal motor seizures onset. Parents reported developmental stagnation after seizure onset followed by progressive regression with loss of language and motor function.

Her clinical exam displayed convergent strabismus, no dysmorphic features. Neurological exam revealed agitation, inability to walk independently, characterized by ataxic gait, poor balance, frequent stumbling. Language skills were limited, with the patient able to say only a few words. Awake EEG showed centro-parietal epileptiform discharges, while the sleep EEG had fronto-central epileptiform discharges. Initial seizure control was obtained using valproic acid, but a relapse occurred after 9 months. Antiseizure medication with phenobarbital and clobazam was initiated but failed. Blood test showed anemia and developmental regression were recorded.

NGS epilepsy gene panel identified a homozygous missense pathogenic variant c.98T > G,p. Met33Arg in *CAD* gene, compatible with EIEE-50.

Uridine 100 mg/kg/day, t.i.d, was started right away. Seizure control was obtained within two days followed shortly by notable psychomotor progress. Improvements included remission of ataxia, ability to walk independently, reduced agitation, advancements in verbal acquisitions and amelioration of anemia. Currently, she is enrolled in a mainstream school, with age-appropriate language ability and full independence in daily activity.

Case 2 and case 3 are sisters from non-consanguineous parents.

Case 2 is the oldest sister, born from an uneventful pregnancy. She was firstly admitted in our clinic at 4 years and 6 months old with epileptic encephalopathy, global developmental delay, and anispoikilocytosis. Seizure onset and neurological progress until first presentation were uncertain, and the patient was previously under medical care abroad. Between 3 and 4 years of age, she had motor and cognitive regression, gradually losing all her abilities. The antiseizure treatment that was administrated to the patient consisted of levetiracetam, phenobarbital, valproic acid topiramate and clonazepam, but with no seizure control. The EEG showed suppression-burst pattern, and the brain MRI revealed cerebral and cerebellar atrophy. Epilepsy gene panel was conducted, but unfortunately, she died a week before the genetic test results arrived.

Case 3 is the youngest sister of case report 2. We firstly evaluated her at 3 years and 5 months, during her sister’s hospital admission. On clinical and neurological exam, she presented cognitive and language deficits, autistic features, and marked agitation. Her verbal communication was limited to only five words, and she had to rely on gestural communication. She did not respond to her name and was able to follow simple commands. Anispoikilocytosis was observed on peripheral blood smear. Seizure onset occurred at 3 years and 6 months old, with bilateral motor aspect. The interictal EEG showed no epileptiform discharges, and an ictal EEG was not obtained.

The results of the epilepsy gene panel showed, in both sisters, the same homozygous pathogenic variant in *CAD* gene, c.98T > G,p.Met33Arg.

Uridine 100 mg/kg/day, administered tree times daily, led to immediate seizure control. Additionally, mild improvements were observed in behavioral status and language including reduced agitation, increased attention span, enhanced eye contact, slight vocabulary expansion, and improved articulation.

Case 4 is a 6-year-old boy from non-consanguineous parents, the third child of the family, born at 32 weeks of gestation with hypoglycemia and hypocalcemia noted at birth. The patient had an older sister with similar phenotype (global developmental delay, intractable epilepsy, blindness) that died during a status epilepticus at 1 year and 4 months. The boy was firstly evaluated in our clinic at 8 months, referred from another clinic. He had seizure onset at 7 months, with upward gaze deviation, hypertonia of the right limbs, with the tendency toward status epilepticus. Multiple antiseizure medications were initiated, such as valproic acid, phenobarbital, and levetiracetam with no control of the seizures. His clinical and neurological exam at 8 months showed global developmental delay, visual impairment, failure to thrive, and a peripheral blood smear that detected anisocytosis with macrocytosis. The EEG showed epileptiform discharges on the right temporal lobal. The brain MRI revealed global cerebral atrophy. During hospitalization, the patient developed recurrent high fever and was diagnosed with sepsis and multiple organ failure and was admitted to the ICU. Whole-exome sequencing testing was conducted. A heterozygous pathogenic variant, c.98T > G,p.Met33Arg, and a heterozygous variant of uncertain significance (VOUS), c.1352A > G,p.Tyr451Cys, in the *CAD* gene were found. As CAD deficiency was suspected and, considering the family history and the severity of the clinical picture, immediately uridine 100 mg/kg t.i.d. was initiated, while the child was still in the ICU, even in the absence of the segregation analysis. Seizure control was obtained in a very short time, and he started improving both motor and cognitive area by regaining his postural control and his ability to grasp object, as well as improving his responsiveness to stimuli.

### 3.2. Clinical Cases in the Framework of Literature Findings

We conducted literature research on PubMed for articles published between January 2010 and March 2024, discussing cases of CAD deficiency. We used as key words “CAD deficiency”, “uridine treatment”, “uridine epilepsy”, “uridine development”. After removing duplicates, articles with no available full-text, articles that were not in English, and articles regarding adult patients, 7 articles remained, with a total of 27 reported patients.

We summarized the clinical findings and symptoms reported and compared them with the findings and symptoms found in our patients (Table 1). One with four cases [22], one with two cases [26], a review with 20 cases that included also the four previous reported cases [27], and five articles with case reports were found [7,17,25,28,29].

## 4. Discussion

EIEE-50 is a rare genetic condition with less than 30 cases reported so far. The findings in our small cohort were similar with those described in the literature. The main clinical features are represented by neurological manifestations, including global developmental delay and drug-resistant epilepsy that were presented in all cases reported before as well as in all our cases. In our cohort, neurological symptoms occurred since the first years of life, having a progressive, degenerative outcome. Firstly, global developmental delay, hypotonia, and/or autism spectrum disorder were observed. Later, drug-resistant epilepsy appears, with polymorphic seizure aspects. Brain MRI is another clue for CAD being a degenerative disease by having the aspect of progressive global brain atrophy. Anisopoikilocytosis is often found and easy to test. Less frequent, the literature describes gastrointestinal symptoms and involvement of the skeleton, heart, and eyes. One of our patients had visual impairment but no other system involvement.

As no bio-marker is available for screening or for follow-up of the deficiency, the genetic testing is the only diagnosis key. The main clinical features suggestive for diagnosis are global developmental delay, drug-resistant epilepsy with psychomotor regression, associated with anemia with anisopoikilocytosis [7].

In our patients, genetic testing detected two variants: the c.98T > G pathogenic variant in homozygous status in the cases 1, 2, 3 and the same variant in heterozygous status compounds with the heterozygous c.1352A > G variant of uncertain significance in the case 4.

The c.98T > G,p.Met33Arg variant replaces methionine, which is neutral and non-polar, with arginine, which is basic and polar, at codon 33 in the carbamoyl phosphate synthetase domain of the CAD protein.

Functional studies on human CAD-knockout cell line have shown that this missense change affects CAD function [30]. Moreover, modeling analyses using CPS I protein showed that the p.Met33Arg change destabilizes subdomain interactions within the CPS2 moiety of CAD, which likely results into altered tertiary protein configuration and consequently a deficient enzymatic function [22]. In silico analysis supports that this missense variant has a deleterious effect on protein structure or function. The variant has been previously described in Bulgarian, Serbian, and Croatian patients with CAD deficiency, highlighting a Balkan-specificity of this allele [22,27].

The c.1352A > G,p.Tyr451Cys) variant replaces tyrosine, which is neutral and polar, with cysteine, which is neutral and slightly polar, at codon 451, which is not located in an established domain, motif, hotspot, or informative constraint region of the CAD protein. In silico analysis supports that this missense variant has a deleterious effect on protein structure/function. Additionally, in silico analysis supports a deleterious effect on splicing. However, this variant has not been described in patients so far and is present in gnomAD < 0.01 for a recessive condition. https://www.ncbi.nlm.nih.gov/clinvar/; https://gnomad.broadinstitute.org/, accessed on 14 November 2024.

The EIEE-50 is now considered as a treatable metabolic condition with poor, dramatic prognosis without an appropriate and an early therapeutic intervention. The administration of Uridine- or Uridine-derivates as soon as is possible is lifesaving [29].

Considering the salvage pathway by exogenous apport of uridine, current treatment offers three options: Triacetyluridine [TAU, uridine triacetate (UT)], UMP (uridine monophosphate), Uridine, with varying degree of efficacity.

Previous reports suggest that uridine at 100 mg/kg/day can lead to rapid seizure control and improvement in cognitive and motor function. UMP, administered at 67–150 mg/kg/day, has shown similar benefits, particularly in resolving anemia and reducing seizures burden, while TAU, despite its higher bioavailability, appears to have more limited effects in some cases.

1. Triacetyluridine (TAU, UT)

Two studies, Refs. [27,28] reported each one patient with CAD deficiency and TAU treatment. Ref. [28]’s patient ceased within 4 days and showed improved mental status. TAU bioavailability of TAU is 4–6 times greater than uridine, requiring lower dosages for treatment, such as 100 mg/kg per day. Ref. [27]’s patient appeared to have a moderate response in seizures; there was a very limited response on GDD, also reporting mild side effect effects including mild nausea, vomiting, or diarrhea.

2. UMP:

One study [27] on 9 patients who were treated with UMP 67 and 150 mg/kg/day, t.i.d. showed important positive effects, as follow: anemia and anisopoikilocytosis resolved in 8 patients; seizure control in 7 patients; 2 patients had seizure control and complete remission of SE; while for 6 patients antiepileptic drugs could be tapered off

3. Uridine: one study including 2 patients treated with oral uridine 100 mg/kg/day [26].

Patient 1 had seizure cession by day 2 and ACDs were tapered off approximately 2-months after, while normalized hemoglobin as well as erythrocytes morphology and nevertheless made significant progress in development. The second patient had prompt cession of seizures by day two, ACDs tapered off approximately 4.5-months after, and there was a dramatic improvement in her psychomotor development, and routine blood tests and the peripheral blood smear both normalized. Koch et al. [22] reported two of the four cases that were treated with uridine, 100 mg/kg uridine. One patient showed cessation of seizures for at least 6 months, normalization of anemia and improvement of gross and fine motor function, as well as cognitive and speech abilities. The other patient, who was in a minimally conscious state with seizures occurring every second day, become awake and showed a quick improvement of alertness and postural control after two months of treatment, along with improvements in auditory, visual, motor, and verbal functions. Only two very short self-limiting seizures in five months of follow up were noted [22]. An immediate positive response on motor and mental function as well as a seizures control were also observed in a patient reported by Peng et al. who was treated with 100 mg/kg/day oral uridine [29].

Response in the patient receiving UMP was generally comparable with those that were treated with uridine. In two cases that were switched from uridine to UMP, no clinical benefits were recorded. Using the TAU therapy is limited by increased costs or by the probability of occurrence of mild side effects. Moreover, only transient improvement in seizures control and minor effects in GDD correction were reported in one patient. However, the superior uridine efficacy in CAD deficiency treatment remains to be investigated [17,27]. Data on long-term efficacy and safety remain scarce, emphasizing the need for further studies to refine the treatment protocols and assess sustained clinical benefits.

Our patients were treated with 100 mg/kg/day uridine administrated in three doses. Seizures control was obtained shortly after the initiation of treatment. The other improvements obtained were in the developmental field including psychomotor, cognitive, language, and behavior. Improvements included the ability to grasp objects, improved responsiveness to stimuli, better postural control, remission of ataxic gait, reduced agitation, increased attention span, enhanced eye contact, improved articulation, vocabulary expansion, and remission of anisopoikilocytosis. Despite the administration of the other treatment for epilepsy, one of the patients included in the study as well as the two other siblings died consequently due to the disease worsening. That result should emphasize the urgency of an early diagnosis and the initiation of the appropriate treatment. Moreover, patient 4 showed a considerable improvement of motor and cognitive skills as well as an immediate control over seizures, which suggests the probability of the deleterious effect of a detected VOUS variant and a trans disposition of the two heterozygous variants.

Our study highlights the importance of early diagnosis and targeted uridine therapy in patients with CAD deficiency-related epileptic encephalopathy. However, given the rarity of this condition and the limited number of patients, larger studies are needed to elucidate its clinical spectrum and optimize treatment approaches. Expanding research to larger, multicenter cohort analyses would help establish standardized diagnosis criteria, assess variability in therapeutic response, and improve long-term outcome prediction. Furthermore, investigating the molecular and biochemical consequence of CAD deleterious modifications could offer deeper insight into the pathophysiology of the disorder. Functional studies using patient-derived cellular models or animal models could clarify the impact of a specific variant on pyrimidine metabolism and neuronal function. Such research will not only enhance our understanding of CAD deficiency but also support the development of targeted therapeutic interventions beyond uridine supplementation.

## 5. Conclusions

EIEE-50 is an autosomal recessive, inborn, progressive, severe metabolic disorder, with poor prognosis in the absence of the specific therapy. Therefore, patients with drug-resistant epilepsy and developmental delay-associating anemia, or with recurrent SE or neonatal seizures, should be tested for CAD deficiency and started on uridine until proven otherwise. No bio-markers are available for the moment, and genetic investigation remains the only diagnostic test.

## Figures and Tables

**Table 1:** Clinical features of our patients compared with those previously reported.

Clinical Features	Our Cohort	Previous Reported Cases	References
Global developmental delay	all 4 cases	18/27	[7,17,22,25,26,27,29]
Drug-resistant epilepsy: multiple seizure types: tonic, generalized tonic–clonic, behavioral arrest, focal motor seizures; +/− fever; tendency to SE	all 4 cases	17/27	[7,17,18,22,25,26,27,28,29]
Regression	case 1, 2, 4	4/27	[22,26,27,28]
Ataxia, tremor	none	3/27	[22,25,27,29]
Hypotonia	case 1,4	5/27	[7,22,25,26,27]
Progressive brain atrophy	case 2,4	13/27	[17,26,27,28]
Autism Spectrum Disorder	case 3	2/27	[27,29]
Hematological involvement abnormal red blood cells (anisopoikilocytosis) and anemia	all cases	8/27	[7,17,22,25,26,27,28]
Gastrointestinal complications: feeding problems, reflux, and recurrent vomiting	none	5/27	[7,17,22,25,27,29]
Less affected systems included the eyes, the skeleton, and the heart	case 1—convergent strabismus case 4—visual impairment	3/27	[22,26,28]

SE—status epilepticus.

## Data Availability

The data that support the findings of this study are available from the corresponding author on reasonable request.

## Abbreviations

The following abbreviations are used in this manuscript:

ATC	Aspartate transcarbamoylase
CAD	Carbamoyl-Phosphate Synthetase 2, Aspartate Transcarbamylase, and Dihydroorotase
CDA	Cytidine deaminase
CDP	Cytidine diphosphate
CMPK	Cytidine monophosphate kinase
CTP	Cytidine triphosphate
CTPS	Cytidine triphosphate synthetase
DEE50	Developmental and epileptic encephalopathy 50
DHO	Dihydroorotase
DHODH	Dihydroorotate dehydrogenase
EEG	Electroencephalogram
EIEE-50	Early infantile epileptic encephalopathy-50
GDD	Global developmental delay
GLN	Glutaminase
ICU	Intensive care unit
NDPK	Nucleoside diphosphate kinase
ODC	Orotidine decarboxylase
OMP	Orotidine monophosphate
OPRT	Orotate phosphoribosyl transferase
PRPP	5-phosphoribosyl-1-pyrophosphate
SE	Status epilepticus
SYN	Carbamoyl phosphate synthetase
TAU	Triacetyluridine
UDP	Uridine diphosphate
UTP	Uridine-5′-triphosphate
UMP	Uridine monophosphate
UMPS	Uridine monophosphate synthase
UT	Uridine triacetate
VOUS	Variant of uncertain significance
